# Comprehensive Pan-Cancer Analysis Identifies POFUT1 as a Prognostic Biomarker and Potential Therapeutic Target Associated with Immune Evasions

**DOI:** 10.3390/cancers18091342

**Published:** 2026-04-23

**Authors:** Zakir Ullah, Xiaosong Pei, Perbhat Ali, Ikram Ullah, Yaqi Li, Shuai Liu

**Affiliations:** 1 Liaoning Provincial Core Lab of Glycobiology and Glycoengineering, Dalian Medical University, Dalian 116041, China; 2 Institute of Cancer Stem Cell, Dalian Medical University, Dalian 116041, China; 3 Department of Biochemistry & Molecular Biology, College of Basic Medical Science, Dalian Medical University, Dalian 116041, China

**Keywords:** POFUT1, fucosylation, tumor immunity, bioinformatics, prognostic biomarker

## Abstract

Background: Aberrant protein O-fucosylation mediated by protein O-fucosyltransferase 1 (POFUT1), has emerged as a hallmark of tumorigenesis that regulates key signaling pathways, including Notch, which is frequently dysregulated in cancers. Protein O-fucosylation, catalyzed by POFUT1, regulates Notch signaling and has been implicated in individual cancers, but its pan-cancer expression patterns, clinical significance, and relationship to tumor immunity remain incompletely characterized. Methodology: We conducted a multi-omics bioinformatics analysis using TCGA and other public datasets to evaluate POFUT1 expression across 33 cancer types (*n* > 10,000). Differential expressions, tumor stage correlations, and survival outcomes were assessed. Immune cell infiltration was estimated using SangerBox and TIMER algorithms, while promoter methylation patterns were analyzed through UALCAN. Functional enrichment and protein–protein interaction networks were constructed to elucidate functional mechanism. Western blot validation in prostate and ovarian cancer cell lines confirmed our computational analysis. Results: POFUT1 showed significant overexpression in 16 of 33 cancer types (FDR-adjusted *p* < 0.05), with the highest elevation in BRCA (*breast invasive carcinoma*; log2FC = 2.31) and LUAD (*lung adenocarcinoma*; log2FC = 2.1). A high POFUT1 expression correlated with poor overall survival in eight cancer types (HR range: 1.8–3.2, *p* < 0.01) and disease-free survival in seven cancers. POFUT1 levels positively correlated with myeloid-derived suppressor cells (MDSCs) infiltrating in 15 cancer types, while inversely correlated with natural killer T (NKT) cells presence in 15 cancers (mean R = −0.34, *p* < 0.05), indicating an association with immunosuppressive microenvironments. Promoter hypomethylation in tumors suggested epigenetic dysregulation as a potential driver of its overexpression. Western blot analysis confirmed POFUT1 protein upregulations in prostate and ovarian cancer cell lines (1.7–2.1-fold. *p* < 0.01), corroborating transcriptomic findings. Conclusion: This pan-cancer study establishes POFUT1 as a critical oncogenic factor linked to aggressive disease, immune evasion, and poor prognosis. Its consistent overexpression and functional impact highlight its potential as a biomarker and target for anticancer therapy. While these computational findings require experimental validation, POFUT1 emerges as a candidate biomarker warranting functional studies and potential therapeutic targeting.

## Simple Summary

Cancer remains a leading cause of death worldwide, and identifying new molecular targets for diagnosis and treatment is critical. POFUT1 is an enzyme that modifies proteins and influences how cells communicate and behave. While previous studies have examined POFUT1 in certain individual cancer types, its role across multiple cancers remains poorly understood. In this study, we analyzed POFUT1 expression patterns, genetic alterations, and clinical outcomes across 33 different cancer types using large-scale patient databases. We found that POFUT1 is abnormally expressed in many cancers and is associated with patient survival, immune cell infiltration, and tumor progression. Our findings suggest that POFUT1 may serve as a potential biomarker for prognosis and a therapeutic target. This comprehensive analysis provides valuable insights into the role of POFUT1 in cancer and may guide future research toward developing POFUT1-targeted therapies.

## 1. Introduction

The molecular pathogenesis of tumorigenesis involves complex interactions among dynamic oncogenic alterations that collectively drive malignant disease progression [1]. This multistep process requires systematic investigation of critical oncogenes to elucidate their roles in carcinogenesis and tumor progression. Large-scale genomic repositories, such as The Cancer Genome Atlas (TCGA), provide invaluable information for analyzing pan-cancer gene expression patterns and functional networks. By leveraging these bioinformatics platforms, we can efficiently characterize oncogene behavior across malignancies, enabling cost-effective, high-throughput identification of potential therapeutic targets and biomarkers [2]. Among post translational modifications, aberrant glycosylation has emerged as a hallmark of cancer, with fucosylation particularly implicated in tumor progression, metastasis, and immune invasion [3]. A crucial regulator of malignant transformation and tumor growth, fucosylation is a significant post-translational alteration that is regulated by fucosyltransferase enzymes. In particular, POFUT1 catalyzes the O-fucosylation of epidermal growth factor (EGF)-like repeats, a change necessary for appropriate Notch receptor folding, trafficking, and functional activation [4]. Notch signaling dysregulation is extensively linked to immunological modulation, cancer start, progression, and metastasis. Given this, POFUT1 is a physiologically significant target for thorough pan-cancer research, offering information on the glycosylation-dependent processes that underlie oncogenic signaling networks [5]. However, systemic pan-cancer characterization of key fucosyltransferases remains limited.

Fucosylation, a major type of glycosylation, is regulated by fucosyltransferases (FUTs). These enzymes catalyze the transfer of fucose residues from the donor substrate, GDP-fucose, to acceptor substrates, including oligosaccharides, glycolipids, and glycoproteins [6]. Early studies demonstrated that FUTs activity is directly proportional to the levels of fucosylated glycans detectable by monoclonal antibodies [7]. Fucose modifies the core of N-glycans by incorporating them into lipid-linked oligosaccharide chains or terminal regions of N- and O-glycans. In some proteins, fucose can also be directly linked to serine or threonine residues. Although N-glycans exhibit considerable structural diversity, they share a common 5-glycocylose core, in which N-acetylglucosamine (GlcNAc) is attached via an amide bond to the asparagine side chain at a conserved consensus sequence.

Protein O-fucosyltransferase 1 (POFUT1, EC 2.4.1.221) is an endoplasmic reticulum (ER) resident glycosyltransferase that catalyzes the O-fucosylation epidermal growth factor-like domains (EGF-LDs). POFUT1 protein comprise a total of 388 AA (amino acid) located in chromosome 20 and features a C-terminal KDEL-like ER retention sequence, having four disulfide bridges and a β-fructosidase-like fold. Structurally, it adopts a GT-B architecture characterized by two Rossmann-fold domains [8]. This modification is essential for Notch receptor function, a pathway frequently dysregulated in cancer [9].

The human genome encodes two protein O-fucosyltransferases: POFUT1 and POFUT2. Despite belonging to the same enzymatic family, these two enzymes have fundamentally distinct and non-overlapping substrate specificities. POFUT1 exclusively modifies EGF-like repeat (EGF-LR) sequences present on Notch receptors (NOTCH1–4) and their ligands (DLL1, DLL4, Jagged1/2), a modification indispensable for Notch receptor folding, trafficking, and signal transduction [8]. POFUT2, in contrast, exclusively modifies O-fucosylates thrombospondin type-1 repeat (TSR) sequences found in structurally unrelated proteins, including ADAMTS proteases, thrombospondins, and complement pathway components, with no overlap with POFUT1 substrates [10]. Because of this complete substrate divergence, functional compensation between POFUT1 and POFUT2 in the context of Notch-dependent oncogenic signaling is structurally precluded. Published transcriptomic evidence further demonstrates that POFUT2 does not exhibit the same consistent pan-cancer upregulation pattern observed for POFUT1 [5], underscoring the enzyme-specific nature of the dysregulation characterized in the present study and justifying the focused investigation of POFUT1.

POFUT1 downregulation has been shown to be lethal in recent studies, as several abnormalities during fetal development in a mouse model have been observed [11]. POFUT1 knocking down has been involved in multiple significant pathological conditions [10]. This protein contributes in development of carcinogenesis by promoting cancer cell cycle, cell proliferation, and cellular invasion [12]. Epithelial–Mesenchymal Transition (EMT) inhibition is caused by downregulated POFUT1 in embryo transfer [13]. Noticeably, the gene alteration of POFUT1 is widely studied in multiple carcinoma, detected in Lung Carcinoma [14], in colorectal carcinoma [15], in bone marrow syndrome [13], and in ovarian carcinoma [16]. Studies showed that Extravascular Trophoblasts (EVTs) overexpress POFUT1, which promotes the HTR/SV neo cell tube formation and transform the endothelial cells of the maternal spiral arterial wall. POFUT1 is involved in co-angiogenesis of trophoblast cells and enhance the potential of HUVECs and HTR/SV neo cells [16].

In early pregnancy, the concentration of epiregulin along with POFUT1 is very high. POFUT1 promotes the production of epiregulin which boost trophoblast EMT on Urokinase-type plasminogen activator [17]. Lung cancer contributes up to 18% of deaths worldwide. POFUT1 and FUT8 mRNA levels were very high in lung cancer patients as compared to normal individuals with sensitivity (81%) and specificity (86%) [18]. Fucosyltransferases exhibit an important role in the investigation of function in the gut microbiome. FUT2 countersigns the typical diversity and composition of gut flora. The higher expression of POFUT2 is associated with gut-associated infections and enhanced disease resistance, and protects the host–microbiome symbiosis [19].

Numerous studies have linked POFUT1 to tumor-promoting mechanisms in individual cancer types [13,14,15,16,17,18]. A pan-cancer analysis by Yu et al. [20] examined POFUT1 as a prognostic predictor with primary emphasis on low-grade gliomas and provided selected immune microenvironment correlations, while Mazour et al. [5] offered a mechanistic review of POFUT1’s oncogenic relevance. However, a systematic, multi-omics pan-cancer characterization integrating POFUT1 expression with the full MDSC–NKT cell immunosuppressive axis, immune checkpoint gene networks, tumor mutational burden (TMB), microsatellite instability (MSI), promoter methylation dynamics, and drug sensitivity data across all 33 TCGA cancer types, supported by protein-level experimental validation, has not been reported. To address these specific gaps, we conducted an integrative pan-cancer analysis of POFUT1 across 33 cancer types, incorporating transcriptomic, proteomic, methylation, mutation, and immune profiling data. Our findings establish POFUT1 as a pan-cancer prognostic biomarker associated with a consistent immunosuppressive microenvironment signature, providing a multi-omics rationale for therapeutic targeting that extends substantially beyond prior published analyses [5,20].

## 2. Methodology

### 2.1. Gene Expression Analysis

Essential data were first gathered from the SangerBox web tool by utilizing TCGA data for a comparison analysis of POFUT1 expression levels within malignant and non-malignant tissues [21]. The list of malignancies is provided in Table 1. However, post-SangerBox evaluation revealed insufficient normal tissue cohorts for numerous malignancies. The web tool GEPIA (http://gepia.cancer-pku.cn/index.html; accessed on 15 November 2024) was incorporated to compare and analyze the expressions of POFUT1 in healthy versus malignant tissues in human cancers [22]. The website TISIDB (http://cis.hku.hk/TISIDB/index.php (accessed on 18th November 2024)) was applied to examine the association between the tumor grade and POFUT1 expression [23]. POFUT1 expressions across normal and metastatic tissues were analyzed using the Kruskal–Wallis test and TNMplot (https://tnmplot.com/analysis/ (accessed on 21 November 2024)) for differential gene expression analysis [24]. The TIMER2 analytical framework was used to assess tumor immune cell infiltration.

Ref. [25] (https://timer.cistrome.org/ (accessed on 24 November 2024)) estimated immune cell abundance from bulk RNA-sequencing data by integrating many well-known computational deconvolution techniques, such as TIMER, CIBERSORT, CIBERSORT-ABS, EPIC, quanTIseq, xCell, and MCP-counter. Specifically, the CIBERSORT and xCell algorithms built within TIMER2 were mainly used to estimate the relative infiltration levels of natural killer T (NKT) cells and myeloid-derived suppressor cells (MDSCs).

The SangerBox platform was used for independent validation in order to increase the robustness of immune infiltration findings (http://sangerbox.com/ (accessed on 27 November 2024)). This uses similar techniques based on deconvolution. Several openly accessible databases were used in tandem to capitalize on their own advantages: SangerBox was used to retrieve TCGA transcriptome data for primary expression analysis; GEPIA (http://gepia.cancer-pku.cn/ (accessed on 27 November 2024)) [22] was utilized to integrate GTEx data with TNMplot to enhance tumor–normal comparisons in cancer types with few normal samples; TNMplot (https://tnmplot.com/analysis/ (accessed on 28 November 2024)) was used with harmonized datasets to evaluate POFUT1 expressions in primary tumor, normal, and metastatic tissues; and TISIDB (http://cis.hku.hk/TISIDB/ (accessed on 29 November 2024)) was especially used to investigate correlations between tumor grade, immune-related characteristics, and POFUT1 expression. Instead of performing direct quantitative cross-platform comparisons, these platforms were utilized for predetermined, complementing analytical reasons. Concordant patterns across independent datasets were viewed as proof of the findings’ robustness, despite the possibility of inter-database heterogeneity due to differences in sample makeup, normalization techniques, and statistical frameworks.

### 2.2. Statistical Analysis and Multiple Testing Correction

The statistical program R (version 4.2.0) was used for all studies. Before hypothesis testing, data distributions were analyzed to choose the best statistical strategy. For comparisons between two groups, Student’s *t*-test was utilized when the data was close to a normal distribution, while for non-normally distributed data or dissimilar sample numbers, the Wilcoxon rank-sum test was employed. The Kruskal–Wallis test was used when there were more than two groups being compared. The non-normal distribution of gene expression data necessitated the use of Spearman’s rank correlation coefficient for correlation analysis. Adjusted *p*-values (q-values) < 0.05 were deemed statistically significant, and the Benjamini–Hochberg technique was used to apply false discovery rate (FDR) correction to account for multiple testing across cancer types, immune cell subsets, and genes.

For all survival analyses, patients within each individual cancer cohort were dichotomized into POFUT1-high and POFUT1-low expression groups using the median expression value of POFUT1 within that cohort as the stratification threshold, which was consistent with established methodology in pan-cancer bioinformatics studies [20]. Kaplan–Meier survival comparisons were performed using the log-rank test (*p* < 0.05). For all immune cell correlation and checkpoint gene analyses, Spearman’s rank correlation with FDR-adjusted q-values (q < 0.05, Benjamini–Hochberg) was used as the significance threshold.

### 2.3. Analysis of Protein Expression by IHC (Immunohistochemistry) Staining

Data from the Clinical Proteomic Tumor Analysis Consortium (CPTAC) was used to investigate tumors and the UALCAN web tool (https://ualcan.path.uab.edu/index.html (accessed on 27 November 2024)) was incorporated to analyze the pattern of POFUT1 expressions between malignant and normal tissue [26]. Additionally, a notable variation was found by UALCAN analysis through the evaluation of IHC images of protein expressions in tumorigenic and healthy samples, followed by the Human Protein Atlas. (https://www.proteinatlas.org/ENSG00000101346-POFUT1/cancer (accessed on 27 November 2024)). The Human Protein Atlas’s immunohistochemical pictures were analyzed not for quantitative comparison but for qualitative visualization of POFUT1 protein expression patterns and localization. No single picture was regarded as typical of consistent protein expression levels, and variations in staining intensity and distribution among the samples were interpreted as indicating inter-individual variability. By comparing tumor and comparable normal tissues using TCGA-derived datasets, the UALCAN platform was used to measure the promoter methylation levels of POFUT1. In this investigation, correlation studies between POFUT1 transcript expression and promoter methylation status were not performed at the sample level.

### 2.4. Prognostic Survival Analysis

Survival outcomes were assessed using GEPIA2 with pan-cancer TCGA cohort analysis generating OS (overall survival) and DFS (disease-free survival) heatmaps for POFUT1. Additionally, Kaplan–Meier (KM) plotter analysis were used for POFUT1’s further prognostic OS and DFS relevance to specific cancer [27]. For each cancer type with significance univariate associations, Cox proportional hazards models were constructed. Hazard ratios (HR) and 95% confidence intervals (CI) were calculated. The proportional hazards assumption was tested using Schoenfeld residuals. Survival analyses were performed using univariate Cox proportional hazards models and Kaplan–Meier calculations, which represent the standard methodological framework for large-scale pan-cancer biomarker discovery analyses utilizing TCGA datasets [20]. Systematic multivariate Cox regression incorporating all clinical confounders (including patient age, sex, tumor stage, grade, and purity) across all 33 cancer types was not performed, as clinical annotation completeness and sample sizes vary substantially between TCGA cohorts, and simultaneous covariate adjustment across all cohorts would compromise statistical power in multiple cancer types. This analytical approach is consistent with published pan-cancer bioinformatics studies of glycosyltransferases and immune-related biomarkers [20,28]. The prognostic survival correlations reported here should accordingly be interpreted as hypothesis-generating associations rather than as evidence of independent prognostic value; confirmatory multivariate analyses within individual cancer types possessing complete clinical annotation represent an important direction for future dedicated studies.

### 2.5. Analysis of Gene Alteration

The web server cBioPortal (https://www.cbioportal.org/ (accessed on 26 November 2024)) was utilized for POFUT1 polymorphism identification [29]. The “TCGA Pan Cancer Atlas Studies” were assigned as a data source, bound by three main parameters (mutation frequency, variant classification, and somatic alteration sites) and were carried out by “TCGA Pan Cancer Atlas Studies” submodules, “Cancer type Summery” and “Mutation interface”. Prognostic correlations between POFUT1 genetic alterations and clinical outcomes (OS/DFS) were evaluated using the “Comparison/Survival” analytical tool in cBioPortal for Cancer Genomics (https://www.cbioportal.org; accessed on 27 November 2024).

### 2.6. POFUT1 Phosphorylation Analysis

Protein phosphorylation, a critical post-translational modification, comprises enzymatic activity and receptor signaling through reversible phosphorylation–dephosphorylation cycles [30]. To investigate its dysregulation in oncogenesis, we performed quantitative phosphorylation profiling of POFUT1 in malignant versus non-malignant tissues by employing the UALCAN data tool, CPTAC.

### 2.7. Assessment of Immune Correlation with POFUT1 Expression

To systematically evaluate the relationship between the expression of POFUT1 and tumor immune microenvironment composition amongst TCGA malignancies, we employed the TIMER2 computational platform for comprehensive immune infiltration profiling [31]. POFUT1 was input into the “Gene” module under the “Immune” section to assess its correlation with immune cell populations. We focused on two functionally opposing immune cell types: MDSCs, which promote tumor progression; and natural killer T cells (NKT), which exert anti-tumor effects. The resulting correlations were visualized using heatmaps and scatter plots. Afterward, we utilized the SangerBox web tool to evaluate associations between POFUT1 and three key oncogenic variables: checkpoints (e.g., PD-1, CTLA-4), TMB, and MSI. These analyses collectively explained the POFUT1 expression in tumor–immune interactions and its implications for cancer progression The main tool used for immune-related correlation analysis was the TIMER2 framework, which takes tumor purity into consideration while creating its computer models. Following independent testing of the major correlation patterns utilizing the SangerBox platform, concordant directional connections across analytical methodologies were viewed as confirming the strength of the reported immunological interactions.

### 2.8. POFUT1 Enrichment Analyses and PPI (Protein–Protein Interactions)

To describe POFUT1-associated protein networks, we integrated complementary bioinformatic platforms, employing the STRING database (https://cn.string-db.org/ (accessed on 2 December 2024)) to identify a systematic map and its interaction partners and functional linkages [32]. By using the STRING database with strict selection criteria, precisely using “Experiments” as the source for interactions and setting a “low confidence” score threshold, we discovered 46 high-probability protein interactors for POFUT1. To authenticate these associations, we carried out complementary analyses in TIMER2.0, utilizing both the “Correlation Analysis” and “Gene_Corr” modules. This dual-module strategy facilitated thorough examination of co-expression patterns across various cancer types through heatmap visualization, as well as a quantitative evaluation of correlation coefficients for the five most significantly interacting genes. All network analyses were performed using the TIMER web server (https://timer.cistrome.org/ (accessed on 2 December 2024)), ensuring the reproducibility of the bioinformatics workflow. Afterward, we accessed the online server (http://bioinformatics.psb.ugent.be/webtools/Venn/ (accessed on 2 December 2024)) to identify the proteins common to both the “POFUT1 interacting” and “POFUT1 correlating” lists. The combined lists were deduplicated, and functional enrichment analysis was conducted using the “enrichplot” and “clusterProfiler” packages [33]. The findings were visualized utilizing the “ggplot2” package in R (version 4.2.0).

### 2.9. Validation Cohort and Experimental Confirmation

Cancer cell lines representing prostate adenocarcinoma (PC3, ATCC CRL-1435; RWPE-1, ATCC CRL-11609) and ovarian carcinoma (SKOV3, ATCC HTB-77; A2780, Sigma-Aldrich Merck KGaA, Darmstadt, Germany, #93112519) were used in this study. PC3, RWPE-1, and SKOV3 cell lines were obtained from the American Type Culture Collection (ATCC, Manassas, VA, USA), and A2780 was purchased from Sigma-Aldrich (Merck KGaA, Darmstadt, Germany), along with normal ovarian surface epithelial cells (IOSE80) as a non-malignant comparator, were selected based on consistent POFUT1 upregulation across TCGA and CPTAC datasets. All cell lines were maintained in RPMI-1640 supplemented with 10% fetal bovine serum (FBS) at 37 °C with 5% CO_2_. IOSE80 cells were obtained from a collaborating laboratory (originally developed by Prof. Nelly Auersperg, University of British Columbia). For Western blot analysis, cells were lysed in RIPA buffer with protease inhibitor cocktail and the total protein was quantified by BCA assay. Equal amounts (15 μg per lane) were resolved on 10% SDS-PAGE gels and transferred to PVDF membranes. Membranes were probed with anti-POFUT1 rabbit polyclonal antibodies (Proteintech Group, Inc., Chicago, IL, USA; Cat. #14929-1-AP; 1:1000) and anti-GAPDH antibodies (Proteintech, Group, Inc. (Rosemont, IL, USA); Cat. #10494-1-AP; 1:5000). Given the known variability of housekeeping proteins across cell types (5, the results are interpreted as qualitative evidence of differential POFUT1 expressions. Two independent biological replicates were performed, each from cells cultured in separate dishes on different days, with full uncropped membrane images for both replicates provided in Supplementary Figure S1. Densitometric quantification was performed using ImageJ software(Version 1.54p; National Institutes of Health, Bethesda, MD, USA).

## 3. Results

### 3.1. Expression Levels of POFUT1 Among Various Tumors

**Figure 1:**
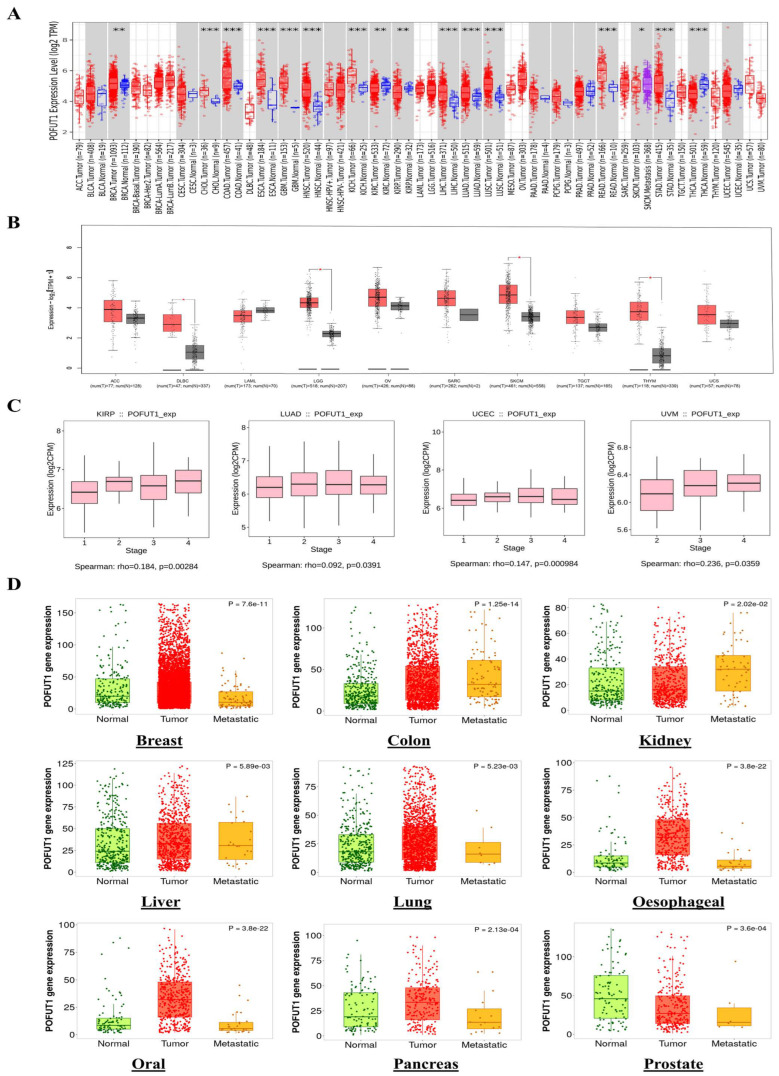
POFUT1 expression profiles in human malignancies. (**A**) TIMER2.0-based assessment of POFUT1 dysregulation in multiple TCGA malignancies; (**B**) POFUT1 expression in additional tumor types lacking matched normal tissue controls, analyzed via GEPIA database; Black: Normal, Red: Tumor (**C**) correlation analysis between POFUT1 expressions and pathological tumor stages across different cancer types using TISIDB web server; and (**D**) comparative analysis of POFUT1 expression between primary tumors and metastatic lesions. Statistical significance: * *p* < 0.05, ** *p* < 0.01, *** *p* < 0.001.

Pan-cancer expression profiling across 33 TCGA cancer types revealed POFUT1 upregulation in 16 malignancies following FDR correction (q < 0.05). Remarkably, POFUT1 exhibited significant overexpression across breast invasive carcinoma (BRCA log2FC = 2.31, q = 1.2 × 10^−45^), lung adenocarcinoma (LUAD log2FC = 2.08, q = 3.1 × 10^−38^), (PRAD, log2FC = 1.64, q = 4.5 × 10^−22^), and liver hepatocellular carcinoma (LIHC log2FC = 1.87, q = 2.4 × 10^−28^) (Figure 1A).

We conducted our analysis with the GEPIA database for cancers whose data were insufficient on the TCGA database. This allowed us to assess the differential expression of POFUT1 between normal tissues and their matched tumor tissues. The results showed significant upregulation of POFUT1 in several malignancies, including lower-grade glioma (LGG), skin cutaneous melanoma (SKCM), diffuse large B-cell lymphoma (DLBC), and thymoma (THYM). In contrast, no significant overexpression was observed in adrenocortical carcinoma (ACC), acute myeloid leukemia (AML), ovarian serous cystadenocarcinoma (OV), sarcoma (SARC), UCS, and testicular germ cell tumors (TGCTs) (Figure 1B).

To assess POFUT1’s potential role in malignant evolution, we analyzed its proteomic profile across different tumor stages using the TISIDB web server. A comparative evaluation of POFUT1 levels in malignant and normal tissues revealed that the correlation was strongly positive in advanced tumor staging within pan-cancer, including KIRP (*p* = 0.0028), UCEC (*p* = 0.00098), UVM (*p* = 0.035), and LUAD (*p* = 0.039). These findings suggest that overexpressed POFUT1 is associated with progression of these malignancies (Figure 1C). Additionally, the TNMplot web server’s module “compare tumor, normal versus metastasis” was employed to estimate POFUT1 mRNA expression levels and tumor occurrence correlations. By investigating the normal and malignant tissues’ results, POFUT1 expression was found to be significantly overexpressed in oral, breast, esophageal, colon, lung, kidney, liver, and pancreatic cancers, except for prostate cancer. The pattern followed by the relationship between tumor and metastatic tissues is described in (Figure 1D). POFUT1 expression patterns were separately analyzed utilizing complementary platforms, such as GEPIA and TNMplot, which incorporate data from TCGA, GTEx, and GEO cohorts, in order to evaluate the robustness of transcriptome findings. The repeatability of POFUT1 deregulation across several cancer types was corroborated by consistent expression patterns found across several different databases. To address the question of potential functional redundancy with its paralogue, it is noted that POFUT2, which exclusively modifies TSR-domain substrates entirely distinct from POFUT1’s EGF-like repeat targets, does not exhibit concordant pan-cancer upregulation in the same cancer types where POFUT1 is overexpressed, as evidenced by the published transcriptomic analyses [5,10,34]. This divergent expression pattern, combined with the completely non-overlapping substrate repertoires of these two enzymes, confirms the specificity and biological relevance of the POFUT1-focused characterization in this study.

### 3.2. Differential Protein Expression

**Figure 2:**
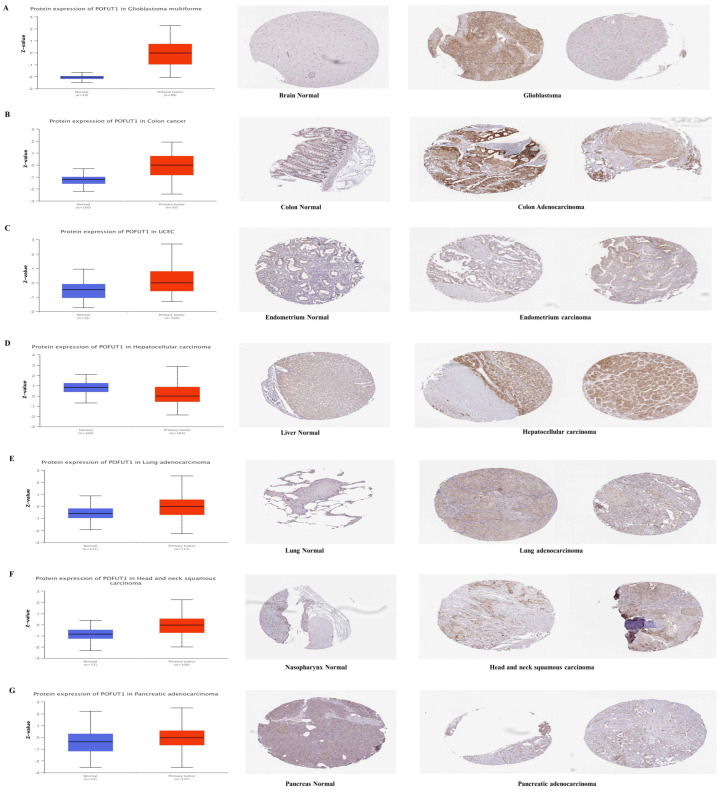
Validation of POFUT1 expressions across multiple tissue types. Box plots demonstrate significantly elevated POFUT1 expressions in tumor tissues (red) compared to adjacent normal tissues (blue) across seven organ systems: (**A**) Glioblastoma Multiforme, (**B**) Colon Adenocarcinoma, (**C**) Endometrial Adenocarcinoma, (**D**) Hepatocellular Carcinoma, (**E**) Lung Adenocarcinoma, (**F**) Nasopharyngeal Carcinoma, and (**G**) Pancreatic Ductal Adenocarcinoma. Immunohistochemistry (IHC) labeling of the tumor sample (**right**) and the normal tissue (**center**) produced consistent results.

Transcriptional and proteomic profiling discovered consistent overexpression of POFUT1 within multiple malignancies. Initially, mRNA-level analysis was carried out by large-scale proteomic investigation using the CPTAC dataset from the NCI (National Cancer Institute). Comparative analysis demonstrated significantly elevated POFUT1 protein expression (*p* < 0.001) in tumor tissues with their matched normal tissues in COAD, UCEC, LUAD, PAAD, GBM, and HNSC cancer tissues. Remarkably, HCC exhibited an inverse expression pattern, with a significantly lower POFUT1 expression compared to normal hepatic tissues (Figure 2A–G). Immunohistochemical validation of these findings showed consistently low-to-moderate staining intensity while significantly elevated, e.g., moderate-to-high staining, intensity in the corresponding malignant tissues.

### 3.3. High POFUT1 Expression Predicts Poor Clinical Outcomes

**Figure 3:**
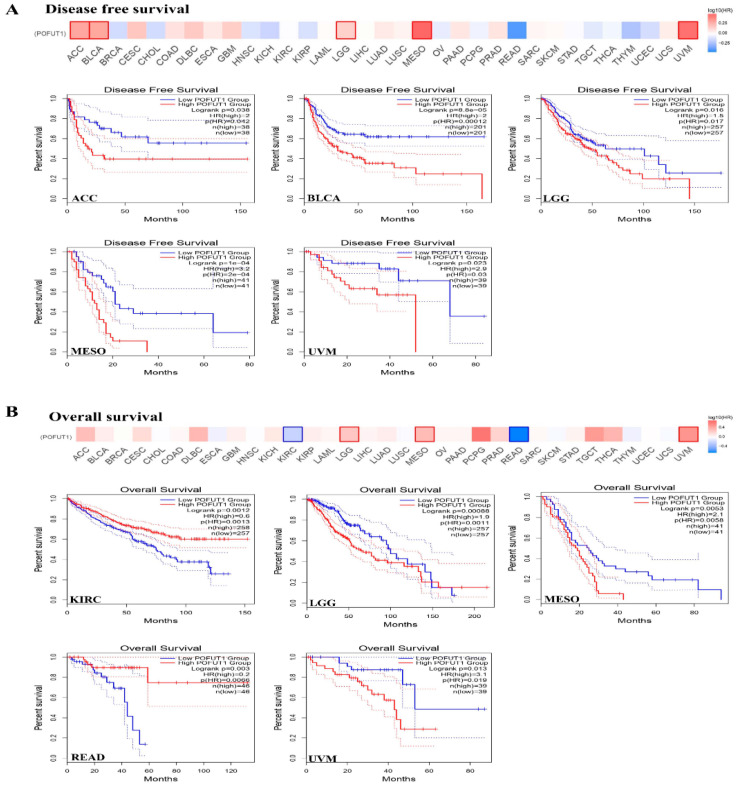
Associations between POFUT1 expression and clinical outcomes in (**A**) disease-free survival and (**B**) overall survival, analyzed using GEPIA. In each survival plot, the *y*−axis demonstrates the survival percentage, while the *x*-axis demonstrates the duration in months. Red curve, high expression; blue curve, low expression.

In all survival analyses, patients within each cancer cohort were stratified into POFUT1-high and POFUT1-low groups using the median POFUT1 expression value as the cutoff threshold, as implemented by the GEPIA2 and KM Plotter platforms. The POFUT1 expression patterns connection with survival rates were carried out by utilizing Kaplan–Meier Plotter (KM Plotter) and GEPIA. Survival analysis across 33 cancer types identified significant associations between high POFUT1 expressions and adverse clinical outcomes in eight malignancies for overall survival (OS) and seven for disease-free survival (DFS). Univariate Cox regression revealed hazard ratios ranging from 1.82 (95% CI: 1.21–2.74, *p* = 0.004, KIRC) to 3.15 (95% CI: 1.82–5.45, *p* = 1 × 10^−4^, MESO) for OS. As per GEPIA analysis, the DFS results for MESO (*p* = 1 × 10^−4^), BLCA (*p* = 8.8 × 10^−5^), ACC (*p* = 0.038), LGG (*p* = 0.016) and UVM (*p* = 0.023), with prognoses associated with a high expression of our target gene (Figure 3A). Moreover, the OS analysis of the patient samples showed a dismal prognosis for LGG (*p* = 0.001), KIRC (*p* = 0.001), MESO (*p* = 0.005), READ (*p* = 0.006), and UVM (*p* = 0.019) (Figure 3B).

**Figure 4:**
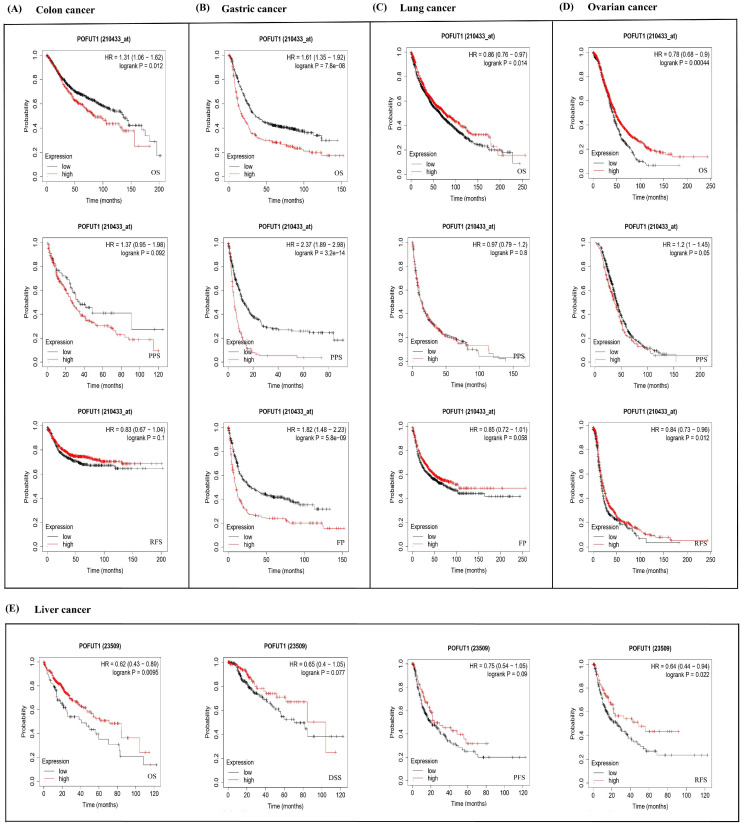
Kaplan–Meier curves evaluating POFUT1-associated survival differences across (**A**) colon, (**B**) gastric, (**C**) lung, (**D**) ovarian, and (**E**) liver malignancies (analyzed via Kaplan–Meier Plotter).

The results of the KM Plotter (https://kmplot.com/ (accessed on29 November 2024)) showed negatively impacted POFUT1 expressions in colon cancer patient OS (*p* = 0.012) (Figure 4A). While in gastric cancer, significant unfavorable correlations in OS (*p* = 7.8 × 10^−8^), Pre-Progression Survival (FPS) (*p* = 5.8 × 10^−9^), and Post-Progression Survival (PPS) (*p* = 3.2 × 10^−14^) were identified in POFUT1 (Figure 4B). Moreover, this gene showed a significant inverse association with OS in lung cancer (*p* = 0.014) (Figure 4C). In OV (ovarian cancer), POFUT1 was studied to have a significant correlation with poor PPS (0.05), OS (*p* = 0.0004), and RFS (Relapse-Free Survival) (*p* = 0.012). Liver cancer (Figure 4D) revealed lower expressions in both RFS (*p* = 0.002) and OS (*p* = 0.009), whereas PPS and DSS were not found to be significant (Figure 4E). These findings reveal generally unfavorable clinical prognoses in cancer patients related to POFUT1 expression patterns. Since the correlations between POFUT1 expression and patient survival were found using univariate analysis, they should be regarded as prognostic correlations rather than proof of standalone prognostic value.

### 3.4. POFUT1 Mutation Burden and Tumorigenesis

**Figure 5:**
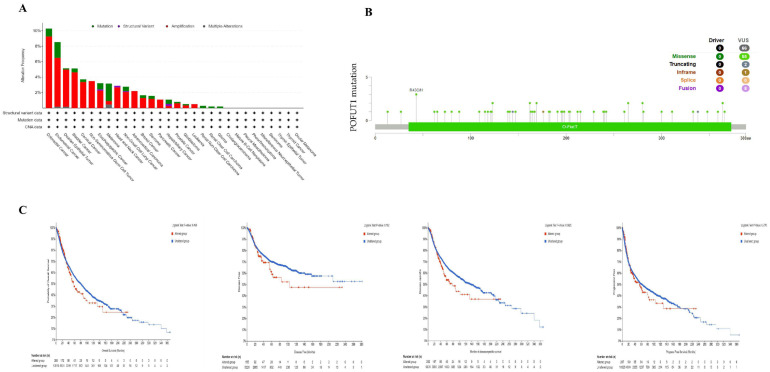
Comprehensive mutation analysis of POFUT1 across human cancers using cBioPortal. (**A**) Alteration frequency of POFUT1 across a panel of human tumor types, displaying various mutation categories; (**B**) distribution and positional mapping of POFUT1 mutation types along the protein structure; and (**C**) survival analysis comparing DSS, DFS, PFS, and OS between POFUT1-altered and unaltered patient groups.

**Figure 6:**
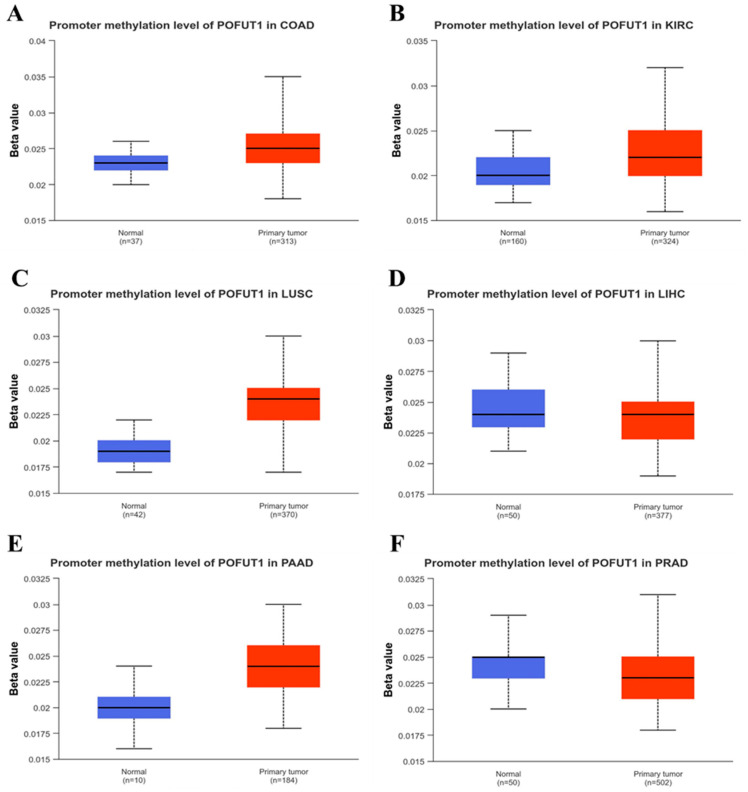
Promoter methylation of POFUT1 in normal versus tumor tissues: (**A**) COAD, (**B**) KIRC, (**C**) LUSC, (**D**) PAAD, (**E**) LIHC, and (**F**) PRAD.

Gene mutations are linked with the most common genetic modifications. According to data from the cBioPortal, mutation analysis has revealed POFUT1 genetic alterations in 3.8% of melanoma cases, with missense mutations predominating (55/66, 83.3%) (Figure 5A). The R43C/H site showed recurrent mutations across colorectal and endometrial carcinomas (Figure 5B). Importantly, mutation status did not significantly correlate with patient survival outcomes (Figure 5C), suggesting POFUT1’s oncogenic role primarily involves transcriptional dysregulation rather than genetic alterations. This finding is consistent with our methylation analysis demonstrating promoter hypomethylation as a driver of POFUT1 overexpression (Figure 6).

### 3.5. Methylation Analysis of POFUT1

DNA methylation analysis was performed to examine the association between POFUT1 promotor methylation status and gene expression. UALCAN database analysis revealed distinct methylation patterns across different tumor types, as promoter hypermethylation was observed in tumor tissues compared to normal tissues in four cancer types: COAD, KIRC, LUSC, and PAAD (*p* < 0.001). Conversely, while promoter hypomethylation was detected in LIHC and PRAD (*p* < 0.01), these showed lower levels of promoter methylation in tumors compared to normal individuals (Figure 6A–F).

### 3.6. Correlation of POFUT1 with Immune Infiltration in Several Tumors

**Figure 7:**
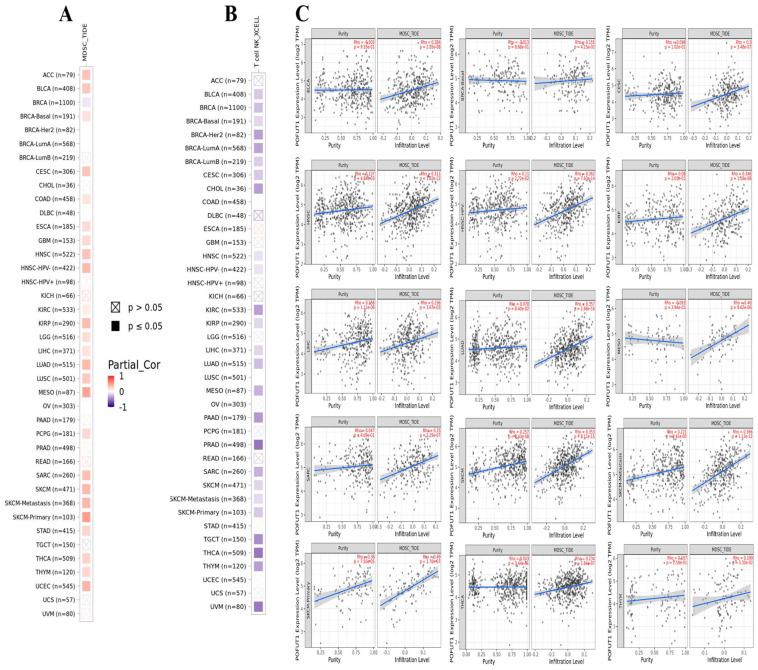
Correlation of POFUT1 expression with immune cell infiltration in human cancers. (**A**) Myeloid-derived suppressor cells (MDSCs), (**B**) natural killer T (NKT) cells, and (**C**) correlation scatter plots of POFUT1 expression and MDSC infiltration levels.

Our investigation of immune cell infiltration patterns revealed significant associations between POFUT1 expression and tumor microenvironment (TME) composition. Analysis of myeloid-derived suppressor cells (MDSCs), known for their immunosuppressive tumor-promoting properties, demonstrated a positive correlation with POFUT1 expression in approximately 60% of TCGA tumors examined. Conversely, an evaluation of natural killer T (NKT) cells, which exhibit potent anti-tumor activity, showed an inverse relationship with POFUT1 expression in over 60% of malignancies (Figure 7). Notably, our comprehensive analysis revealed no instances of negative correlations between MDSC infiltration and POFUT1 expression, nor any positive associations with NKT cell infiltration across the tumor panel. Through stringent filtering criteria, we identified 15 specific tumor types, including THYM, THCA, SKCM (both primary and metastatic), SARC, MESO, LUAD, LIHC, KIRP, HNSC (both HPV+ and HPV−), CESC, BRCA, and BLCA, that consistently exhibited dual correlations, e.g., significant positive association between POFUT1 expression and infiltrated MDSCs, coupled with negative correlation with NKT cell presence (Figure 7A,B). Together, our findings suggest that an elevated POFUT1 expression is associated with an immunosuppressive tumor microenvironment in a variety of cancer types, as seen by a decreased natural killer T cell abundance and an increased infiltration of myeloid-derived suppressor cells (Figure 7C).

**Figure 8:**
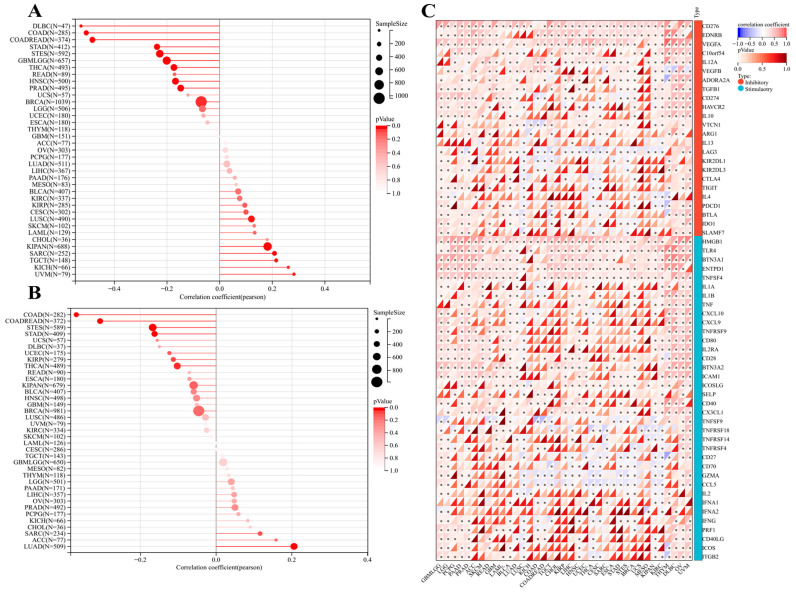
POFUT1 stimulatory and inhibitory correlations with immune checkpoints: (**A**) MSI; (**B**) TMB by bar plot. (**C**) Correlation of immune checkpoints and POFUT1 expression across listed human malignancies shown by a heatmap (∗: *p* < 0.05).

Moreover, SangerBox was used to further elucidate the immune checkpoint correlation with POFUT1 expression in SARC, TGCT, KICH, KIPAN, CHOL, AML, SKCM, and UVM tumors, showing a significant positive association with microsatellite instability (MSI), while analysis of tumor mutational burden (TMB) revealed a strong positive correlation with POFUT1 expression in LUAD, ACC, SARC, CHOL, KICH, PCPG, PRAD, OV, LIHC, and PAAD (Figure 8A,B). Additionally, about 60 stimulatory and inhibitory immune checkpoint genes showed a significant positive correlation with POFUT1 expression in MESO, KIPAN, and CHOL, suggesting a potential association with immune checkpoint regulation. In contrast, tumors such as LUSC, THYM, and TGCT exhibited no significant correlation between POFUT1 expression and most immune checkpoint genes. (Figure 8C).

### 3.7. PPI and Enrichment Analysis of POFUT1

**Figure 9:**
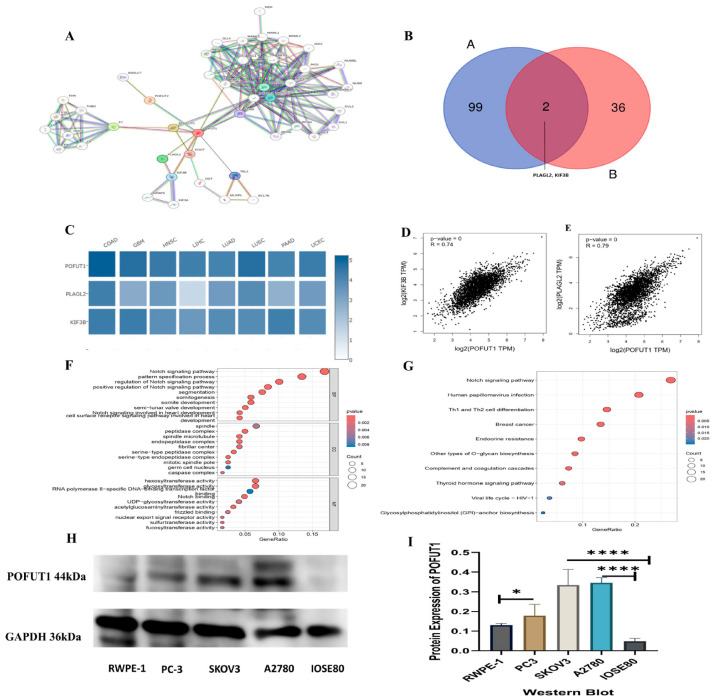
Protein network interaction and functional enrichment analysis of POFUT1. (**A**) STRING database-derived interaction network displaying the top 46 proteins with highest interaction confidence scores for POFUT1; (**B**) Venn diagram illustrating the overlap between POFUT1-interacting proteins and correlating genes among cancer types; (**C**) heatmap depicting expression patterns of two commonly correlated genes (PLAGL2 and KIF3B) POFUT1 across multiple cancer types; (**D**,**E**) correlation analyses of POFUT1 expressions with KIF3B (R = 0.79) and PLAGL2 (R = 0.74); (**F**) GO enrichment analysis of POFUT1-interacting and binding partners, showing enriched BP, CC, and MF as visualized bubble plots; (**G**) KEGG pathway enrichment analysis presented as a bubble plot illustrating the involvement of POFUT1-associated genes in key signaling pathways; (**H**) Western blot confirmation of POFUT1 protein expression in prostate adenocarcinoma (RWPE-1, PC-3) and ovarian carcinoma (SKOV3, A2780, IOSE80) cell lines, as well as showing POFUT1 (~44 kDa) and GAPDH (~36 kDa); and (**I**) densitometric quantification of POFUT1 protein expression normalized to GAPDH. Data are presented as mean ± SD from two independent biological replicates. * *p* < 0.05; **** *p* < 0.0001. The uncropped blots are shown in Figure S1.

Our results linking POFUT1 to immune modulation and poor prognosis prompted investigation of its molecular pathways. Using STRING and GEPIA2, we identified 46 protein interactors and 100 co-expressed genes, respectively (Figure 9A). Venn analysis revealed two shared genes, PLAGL2 and KIF3B, which showed significant positive correlation with POFUT1 across TCGA tumors (Figure 9B). These conserved interactors suggest key mechanistic roles for POFUT1 in tumor progression. The heatmap signifies the positive correlation between the interacting genes PLAGL2 and KIF3B (Figure 9C). The GEPIA2.0 web server was explored for the relative correlation expression, and their expression correlation was KIF38 = 0.74 and PLAGAL2 = 0.79 (Figure 9D,E).

Integrated functional enrichment analysis of the combined gene lists (duplicates removed) was performed using R packages “clusterProfiler” and “enriched plot”. Biological processes related to Notch signaling, embryonic patterning, segmentation, and cardiac cell surface receptor-mediated signaling were significantly enriched among genes associated with POFUT1 expression, according to gene ontology enrichment analysis. Molecular analysis identified glycosyltransferase and hexosyltransferase activities (Figure 9F). These genes were subsequently shown to be substantially linked to several signaling pathways involved in developmental control, cell fate determination, and carcinogenic activities, including Notch signaling, human papillomavirus infection, breast cancer, Th1/Th2 cell differentiation, and O-glycan biosynthesis, by KEGG pathway enrichment analysis (Figure 9G). It should be noted that co-expression patterns and well-maintained pathway databases, rather than direct functional experiments, are the sources of the pathway enrichment and protein–protein interaction studies carried out in this work. Therefore, the goal of these studies was not to establish causal linkages but rather to offer biologically plausible possibilities. A possible functional significance that warrants additional experimental confirmation is indicated by the observed abundance of Notch signaling, immune-associated pathways, and processes connected to the epithelial–mesenchymal transition among genes correlated with POFUT1 expression. To validate POFUT1 expression at the protein level, Western blot analysis was performed across multiple cancer cell lines and normal controls. POFUT1 protein was markedly elevated in prostate adenocarcinoma (PC-3 vs. RWPE-1) and ovarian carcinoma (SKOV3 and A2780 vs. IOSE80) cell lines (Figure 9H). Densitometric quantification confirmed consistent POFUT1 upregulation in cancer lines relative to their respective normal controls (Figure 9I). Western blot validation confirmed POFUT1 protein upregulation in prostate (PC-3 vs. RWPE-1, *p* = 0.003) and ovarian (SKOV3: 2.1-fold; A2780: 1.7-fold vs. IOSE80, *p* < 0.01) cancer models, corroborating TCGA transcriptomic data (Figure 9H,I).

## 4. Discussion

This systematic pan-cancer analysis establishes POFUT1 as a clinically relevant biomarker with consistent associations across multiple malignancies. By integrating transcriptomic, proteomic, epigenetic, and immunological data from over 10,000 tumor samples across 33 cancer types, we demonstrate that POFUT1 overexpression: (1) occurs in approximately half of solid tumor types; (2) correlates with adverse survival in eight cancers; (3) associates with immunosuppressive microenvironments characterized by MDSC enrichment and NKT cell depletion; and (4) results primarily from epigenetic dysregulation rather than genetic mutations. These findings provide compelling rationale for functional validation studies and therapeutic targeting strategies.

Fucosylation, a critical post-translational modification, catalyzed by fucosyltransferases like POFUT1, regulates cell signaling, adhesion, and immune responses [35]. Fucosylated glycans such as sialyl-Lewis X serve as biomarkers for aggressive cancer phenotypes and have been implicated in tumor progression, metastasis, and poor prognosis [36]. Global or site-specific fucosylation in tumor tissues was not explicitly evaluated in this investigation, despite the fact that POFUT1 expression showed consistent dysregulation across a variety of cancer types. As a result, changes in fucosylation were not empirically confirmed at the biochemical level but rather deduced from levels of enzyme expression. Therefore, in tumors with increased POFUT1 expression, functional glycosylation alterations will need to be confirmed by direct measures of fucosylation. Significant correlations between POFUT1 expression and genes within the Notch–HES1–STAT3 signaling axis suggest a possible connection to Notch-related pathways found by enrichment studies. Increased concentration of M2-like macrophages and immunosuppressive tumor features have been linked to enhanced POFUT1 expression in glioblastoma multiforme (GBM) [20]. In addition to Notch signaling, functional enrichment analyses revealed a number of other biological pathways that may be connected to POFUT1 dysregulation. These include O-glycan biosynthesis, processes associated with the epithelial–mesenchymal transition, immune differentiation pathways like Th1/Th2 cell differentiation, and more general signaling networks related to cancer. These enhanced pathways align with the known functions of abnormal glycosylation in immunological regulation, tumor growth, migration, and cell adhesion. Although Notch signaling was given priority because of its direct reliance on O-fucosylation and POFUT1’s known enzymatic activity, the presence of these other pathways indicates that POFUT1 may have a variety of context-dependent effects on tumor biology. It will need focused functional and mechanistic research to clarify the relative contributions of these pathways across various cancer types. All of these findings point to POFUT1 as a possible therapeutic target and prognostic biomarker, pending more functional validation to elucidate its involvement in tumor–immune modulation.

However, its comprehensive impact across cancer types, particularly regarding TME interactions and immune modulation, remains underexplored. The current study was carried out across multiple cancer types to overcome this gap. Our findings extend previous single-cancer studies of POFUT1 by revealing common patterns across malignancies, while Zhang et al. demonstrated POFUT1’s pro-metastatic role in HCC specifically [12]. Importantly, the scope and analytical depth of the present study differ substantially from prior published pan-cancer analyses of POFUT1. Yu et al. [20] examined POFUT1 prognostic significance with a primary focus on low-grade gliomas and provided selected immune correlations. The present study extends these findings across all 33 TCGA cancer types through five specific contributions not reported in that work: (1) systematic profiling of the dual MDSC–NKT cell immunosuppressive axis across the full TCGA pan-cancer spectrum using two independent deconvolution platforms (TIMER2.0 and SangerBox), establishing a consistent pan-cancer immune evasion signature; (2) comprehensive integration of immune checkpoint gene correlation data (~60 stimulatory and inhibitory genes) across cancer types; (3) integrated characterization of POFUT1 associations with TMB and MSI as potential predictive biomarkers for immunotherapy response; (4) identification of PLAGL2 and KIF3B as novel POFUT1-correlated protein interaction partners with mechanistic relevance to Notch-driven tumor biology; and (5) protein-level experimental validation through Western blot analysis in prostate and ovarian cancer cell line models, directly corroborating computational findings. Together, these contributions represent a substantially more comprehensive multi-omics characterization of POFUT1 in cancer than previously reported. Our pan-cancer approach identifies POFUT1 as a broadly relevant oncogenic factor. The consistency of POFUT1 upregulation across histologically diverse cancers suggest convergent evolution toward Notch pathway dependence during oncogenesis. Recent pan-cancer analyses of other glycosyltransferases support this model. For example, FUT8 shows similar pan-cancer overexpression patterns correlating with poor prognosis [28], while B3GNT3 exhibits context-dependent oncogenic or tumor-suppressive roles [37].

Our POFUT1 findings align with emerging recognition that aberrant glycosylation represents a targetable cancer hallmark.

Survival analysis serves as a fundamental analytical approach for evaluating disease outcomes and therapeutic efficacy in clinical research [38]. Platform-specific partial heterogeneity was seen in POFUT1 survival studies using GEPIA and the Kaplan–Meier Plotter. Given that the Kaplan–Meier Plotter incorporates several GEO-based cohorts, while GEPIA predominantly uses TCGA–GTEx data, these discrepancies most likely result from differences in underlying datasets, cohort makeup, survival criteria, and analytical methods. Therefore, rather than being seen as contradictory data on the prognostic significance of POFUT1, these platform-dependent differences should be seen as context-specific results. Mutations in several genes were found to be good prognostic markers for human cancer, with examples including mutated KRAS that was correlated with a poor prognosis of pancreatic cancer [39] and lung cancer [40], as well as mutated NRAS that was associated with a poor prognosis of metastatic melanoma [41]. Importantly, the survival relationships shown here were derived from univariate Cox proportional hazards and Kaplan–Meier analyses and did not take into consideration recognized pathological or clinical prognostic factors, such as tumor purity, histological grade, tumor stage, or patient age. Therefore, these results should not be seen as proof of independent prognostic relevance, but rather as prognostic associations. Whether POFUT1 adds prognostic value beyond traditional risk markers will require further research that includes multivariate survival models within specific cancer types, especially those with thorough clinical annotation. Regarding the potential influence of demographic confounders, it is relevant to note that TCGA pan-cancer cohorts were assembled using standardized case selection protocols with age and sex distributions representative of the respective cancer populations [34]. The TIMER2.0 platform, employed for immune infiltration analysis, incorporates tumor purity as a covariate in its computational deconvolution models, partially correcting for tumor cellularity-related confounding in immune cell estimates [25]. Furthermore, the biological mechanism through which POFUT1 may influence prognosis, namely, O-fucosylation-dependent Notch receptor activation driving MDSC recruitment and NKT cell suppression, represents a cell-intrinsic oncogenic pathway whose operation is not contingent on patient age or sex. While dedicated multivariate adjustments for demographic variables within individual cancer types with complete clinical annotation are necessary steps before clinical translation, the mechanistic coherence of POFUT1’s associations across multiple independent analytical platforms provides biological plausibility that transcends demographic confounding. Therefore, our next survival analysis step was to study whether the POFUT1 genetic alteration could also affect patient’s survival: we found that POFUT1 genetic alteration did not significantly correlate with the patient’s survival in four analyzed models of altered and unaltered groups.

DNA methylation dynamics have been well-characterized across multiple malignancies, with hypermethylation established as a critical epigenetic mechanism driving transcriptional silencing of tumor suppressor genes, particularly through CpG island promoter modification [42]. Examples include ELMO3, AQP1, and LINE-1 hypermethylation observed in lung cancer [43], salivary gland adenoid cystic carcinoma [44], and colorectal cancer [44]. In contrast to this paradigm, our POFUT1 methylation analysis revealed hypomethylation in COAD, KIRC, LUSC and PAAD tumors while LIHC and PRAD exhibited hypermethylation compared to normal tissues. Despite the fact that POFUT1 promoter methylation changes were seen in a variety of cancer types, these patterns varied, with both hypermethylation and hypomethylation seen based on the tumor setting. This suggests that POFUT1’s epigenetic regulation is particular to cancer and not controlled by a single, overarching mechanism. DNA methylation and gene expression have a very context-dependent connection, even though promoter hypomethylation is frequently linked to transcriptional activation. Although it does not constitute a universal regulatory mechanism, promoter hypomethylation may be a contributing factor to increased POFUT1 expression in some cancers, according to this study. Interestingly, there were no clear negative associations between promoter methylation and POFUT1 expression in other malignancies, including lung squamous cell carcinoma (LUSC) [40]. Given that the position of the CpG probe, tissue-specific chromatin states, transcription factor activity, and other epigenetic or genomic regulatory processes all affect the functional impact of DNA methylation, this discrepancy is scientifically believable.

Tumor immunotherapy has emerged as a pivotal anti-cancer strategy in recent decades [45], with immune checkpoint inhibitors such as αPD-1 demonstrating efficacy in HCC [46]. To explore this further, we examined the correlation between elevated POFUT1 expression and immune cells infiltration in tumors. Notably, MDSCs (myeloid-derived suppressor cells) positively correlate with metastasis and tumor cell survival [47]. MDSCs suppresses anti-tumors immunity by inhabiting NK and CD8 T cells, promoting tumor angiogenesis and metastatic stem cells [21]. Cytokines including CSF1, CCL5, and CCL2 mediate MDSC recruitment for tumor sites [48]. Our analysis revealed positive correlation between POFUT1 expression and MDSC infiltration in BLCA, CESC, ACC, SKCM, GBM, HNSC, LUAD, MESO, UCS, SARC, and KIRP. However, this observed correlation between POFUT1 expression and MDSC infiltration warrants further investigation.

PARP1 upregulation may mechanistically link to specific chemokine expression patterns, potentially explaining the observed association. Conversely, natural killer T (NKT) cell infiltration, recognized for its tumor-suppressive functions, emerged as a favorable prognostic indicator across multiple malignancies, correlating with improved survival outcomes [49]. Present study exhibited a notable negative correlation between NKT infiltration and POFUT1 expression in, TGCT, BLCA, CHOL, LIHC, KIRC, PAAD, PRAD, THCA, THYM, and UVM. Remarkably, no tumors in our dataset exhibited a positive correlation between POFUT1 expression and NKT cell infiltration. Integrated analysis of POFUT1’s inverse associations with NKT cells and positive links to MDSC infiltration suggests that elevated POFUT1 levels may signify impaired anti-tumor immunity, potentially serving as a biomarker for immune evasion. Strong correlations between POFUT1 expression, increased myeloid-derived suppressor cell infiltration, and decreased natural killer T cell abundance were found in the current analysis; however, these findings are correlative and do not prove causality. Myeloid cell recruitment and NKT cell exclusion in the tumor microenvironment are regulated by downstream cytokine and chemokine signaling pathways, which may be influenced by POFUT1-dependent O-fucosylation of Notch receptors. Targeted functional investigations, such as POFUT1 gain- and loss-of-function models, in conjunction with immune co-culture systems or in vivo tumor models, will be necessary for the direct evaluation of these possibilities.

Immune evasion may be facilitated by POFUT1-mediated O-fucosylation via a variety of non-exclusive mechanisms. Proper Notch receptor folding, maturation, and signaling depend on O-fucosylation, which POFUT1 catalyzes. Notch activity has been linked to the alteration of cytokine and chemokine networks that promote the recruitment of myeloid-derived suppressor cells and hinder the function of cytotoxic lymphocytes. Furthermore, in the tumor microenvironment, immune cell trafficking, ligand–receptor interactions, and immune identification can all be impacted by abnormal glycosylation of cell-surface receptors. These processes provide physiologically believable explanations for the observed correlations between increased POFUT1 expression, improved MDSC penetration, and decreased natural killer T cell abundance, despite the fact that they were not examined explicitly in this investigation.

TMB and MSI have been established as predictive biomarkers for immunotherapy efficacy in cancer patients, with studies demonstrating their prognostic value in clinical outcomes [50]. A robust anti-tumor response to αPD1 therapy was notably observed in CRC patients exhibiting high microsatellite instability. Similarly, elevated tumor mutational burden (TMB) was consistently associated with improved clinical outcomes across diverse tumor types [51]. Our analysis revealed POFUT1 overexpression in tumor tissues correlates with MSI in multiple malignancies (COAD, HNSC, GBM, LUSC, LIHC, LUAD, PAAD, UCEC) and TMB, suggesting its potential as a predictive biomarker for immunotherapy response. Finally, to elucidate its mechanistic role, we identified POFUT1-associated protein networks, highlighting pathways central to tumor progression and immune modulation. Based on co-expression patterns and analysis of protein–protein interaction networks, proteomic investigations revealed that PLAGL2 and KIF3B were the proteins most significantly associated with POFUT1. According to earlier research, PLAGL2 promotes the migration and proliferation of cancer cells, whereas KIF3B plays a role in cell motility and vesicular transport. Nevertheless, there is still no experimental evidence of a possible functional connection between POFUT1 and these proteins, which calls for more research [19,52]. The oncogenic roles of PLAGL2 and KIF3B remain inadequately characterized. Given their strong correlation with POFUT1 in tumor tissues, elucidating their cancer-driving mechanisms and interaction networks is essential. Such investigations could yield valuable insights and inform the development of novel therapeutic strategies in cancer treatment.

Western blot analysis confirmed elevated POFUT1 protein expression in prostate adenocarcinoma cell lines (PC-3 vs. RWPE-1; *p* = 0.003) and ovarian carcinoma lines (SKOV3: 2.1-fold; A2780: 1.7-fold; both vs. IOSE80, *p* < 0.01). These models were selected based on robust POFUT1 upregulation across both TCGA and CPTAC datasets, representing cancer types with consistent transcriptomic and proteomic evidence of POFUT1 dysregulation. The concordance between mRNA abundance (TCGA), protein quantification (CPTAC), and direct Western blot measurement across three independent data layers provides multi-platform corroboration for POFUT1 overexpression in these malignancies and validates the transcription-level basis of this dysregulation. Hepatocellular carcinoma, which exhibited context-dependent POFUT1 expression patterns likely reflecting etiological heterogeneity (HBV vs. HCV vs. metabolic disease backgrounds), was not subject to in vitro Western blot validation in the current study owing to the absence of a verified matched hepatic normal cell line, which is acknowledged as a limitation.

Our experimental validation lays the foundation for future mechanistic studies. Differences in POFUT1 expression across cancer types suggest a context dependence of oncogenic functions. In prostate and ovarian cancers with elevated POFUT1, studies should focus on Notch pathway activation, cancer stem cell maintenance, and chemotherapy resistance. In hepatocellular carcinomas with variable expression, studies should examine whether alternative fucosylation pathways compensate for POFUT1 downregulation or whether these reflect different molecular subtypes with different therapeutic vulnerabilities. Combining bioinformatics analysis with experimental validation allows computational predictions to be directly confirmed at the molecular level, reinforcing the reliability of the findings. Experimental validation was performed in prostate adenocarcinoma (PC-3, RWPE-1) and ovarian carcinoma (SKOV3, A2780, IOSE80) cell lines, which were selected based on robust and reproducible POFUT1 upregulation signals across both TCGA and CPTAC datasets. Western blot analysis confirmed elevated POFUT1 protein expression in cancer lines relative to their normal controls in both cancer types, corroborating the bioinformatic findings. TCGA and CPTAC computational data for hepatocellular carcinoma remain in the manuscript; however, in vitro Western blot validation of LIHC was not performed in this study owing to the absence of a verified hepatic normal cell line, which is acknowledged as a limitation.

### 4.1. Clinical and Therapeutic Implications  

The clinical implications of our findings are multifaceted. First, POFUT1 emerges as a potential pan-cancer prognostic biomarker with a hazard ratio (1.8–3.2) comparable to established marker like Ki-67. Second, the association with immunosuppressive microenvironments suggests POFUT1-high tumors may respond poorly to immune checkpoint inhibitors. Third, there are presently no selective inhibitors that target POFUT1, despite the fact that the enzyme’s enzymatic activity suggests possible therapeutic significance. Therefore, before the viability of POFUT1 as a therapeutic target can be determined, thorough functional validation will be required.

The identification of PLAGL2 and KIF3b as highly correlated partners provides mechanistic hypotheses. PLAGL2 is a zinc finger transcriptional factor that promotes EMT and metastasis in gastric cancer [19], suggesting POFUT1 may regulate cancer stem cell programs. KIF3B is a kinesin motor protein involved in intraflagellar transport and cilia formation, which regulates hedgehog signaling pathways [53], and exhibits crosstalk with Notch signaling in cancer development. These connections warrant direct experimental investigation.

### 4.2. Limitations of the Study

Our study has important limitations. The analyses relied primarily on computational methods with limited experimental validation, requiring functional studies to establish causality. Immune cell infiltration estimates were derived from deconvolution algorithms rather than direct quantification. TCGA cohorts represented treatment-naive primary tumors, leaving POFUT1’s role in treatment resistance unexplored. Future studies should prioritize functional validation using CRISPR approaches, mechanistic investigations of POFUT1–immune interactions, clinical validation in independent cohorts, and single-cell sequencing to elucidate cell-type-specific expression patterns.

It should be noted that this study has a number of limitations. Computational deconvolution techniques were used to predict immune cell infiltration from bulk RNA-sequencing data obtained using TIMER2 and SangerBox. These methods are often employed in pan-cancer investigations and have received extensive validation; nonetheless, they are not able to adequately capture the spatial organization of immune cells within the tumor microenvironment, stromal contamination, or tumor purity effects. Even though TIMER2 accounts for partial tumor purity correction, estimations of immune infiltration are still indirect and should be used with caution. To better define the relationship between POFUT1 expression and immune composition, future validation utilizing flow cytometry, immunohistochemistry, single-cell RNA-sequencing, and spatial transcriptomic approaches will be necessary because tumor immunity is intrinsically cell-type-specific and spatially structured.

Additionally, rather than utilizing quantitative validation, the assessment of protein expression using Human Protein Atlas immunohistochemistry pictures was qualitative and relied on publicly accessible datasets, offering contextual support. Quantitative scoring in separate patient cohorts and standardized immunostaining will be necessary for thorough validation of POFUT1 protein expression. Lastly, only two cancer types (prostate and ovarian) were included in the experimental validation by Western blot. This supports bioinformatic expression trends in specific contexts, but it does not fully capture the biological heterogeneity across all tumor types examined, and it should not be interpreted as universal validation.

## 5. Conclusions

This pan-cancer analysis establishes POFUT1 as significant oncogenic factor with clinical potential. In all 33 types of cancer, higher POFUT1 expression was consistently linked to advanced disease stage, poor patient outcomes, and an immunosuppressive tumor microenvironment characterized by fewer natural killer T cells and more myeloid-derived suppressor cells infiltrating the tumor. Its association with TMB and MSI suggested its potential dual utility as both a prognostic indicator and predictor of immunotherapy response. Additionally, POFUT1 dysregulation was linked to alterations in promoter methylation in a way unique to the type of cancer, with hepatocellular carcinoma exhibiting especially context-dependent expression patterns. Western blot validation in prostate and ovarian cancers computational predictions, while protein network analysis linked POFUT1 to Notch signaling through partners PLAGL2 and KIF3B. Even though this work relied on retrospective database analysis and had limited experimental validation, it offers compelling evidence that POFUT1 is a predictive biomarker and a potential therapeutic target, deserving of more functional and molecular research. Future efforts should focus on developing POFUT1-targeted therapies, validating its clinical utility in prospective trials, and exploring combination strategies with immunotherapy to improve patient outcomes across diverse malignancies.

## Figures and Tables

**Table 1:** The abbreviations and full names of analyzed tumors in the current study.

Abbreviation	Tumor Name
ACC	Adrenocortical carcinoma
BLCA	Bladder urothelial carcinoma
BRCA	Breast invasive carcinoma
CESC	Cervical squamous cell carcinoma and endocervical adenocarcinoma
CHOL	Cholangiocarcinoma
COAD	Colon adenocarcinoma
DLBC	Lymphoid neoplasm diffuse large B-cell lymphoma
ESCA	Esophageal carcinoma
GBM	Glioblastoma multiforme
HNSC	Head and neck squamous cell carcinoma
KICH	Kidney chromophobe
KIRC	Kidney renal clear cell carcinoma
KIRP	Kidney renal papillary cell carcinoma
AML	Acute myeloid leukemia
LGG	Lower grade glioma
LIHC	Liver hepatocellular carcinoma
LUAD	Lung adenocarcinoma
LUSC	Lung squamous cell carcinoma
MESO	Mesothelioma
OV	Ovarian serous cystadenocarcinoma
PAAD	Pancreatic adenocarcinoma
PCPG	Pheochromocytoma and paraganglioma
PRAD	Prostate adenocarcinoma
READ	Rectum adenocarcinoma
SARC	Sarcoma
SKCM	Skin cutaneous melanoma
STAD	Stomach adenocarcinoma
TGCT	Testicular germ cell tumor
THCA	Thyroid carcinoma
THYM	Thymoma
UCEC	Uterine corpus endometrial carcinoma
UCS	Uterine carcinosarcoma
UVM	Uveal melanoma

## Data Availability

The data presented in this study are available from publicly accessible databases. Transcriptomic data were obtained from TCGA via TIMER2.0 (https://timer.cistrome.org/ (accessed on 24 November 2024)), GEPIA2 (http://gepia2.cancer-pku.cn/ (accessed on 27 November 2024)), and TNMplot (https://tnmplot.com/ (accessed on 28 November 2024)). Proteomic data were accessed via UALCAN/CPTAC (https://ualcan.path.uab.edu/ (accessed on 27 November 2024)). Survival data were retrieved from KM Plotter (https://kmplot.com/ (accessed on 29 November 2024)) and GEPIA2 (accessed on 29 November 2024). Mutation data were obtained from cBioPortal (https://www.cbioportal.org/ (accessed on 26 November 2024)). All tools and databases are freely accessible.

## Supplementary Materials

The following supporting information can be downloaded at: https://www.mdpi.com/article/10.3390/cancers18091342/s1, Figure S1: Full uncropped Western blot membrane images from two independent biological replicates confirming POFUT1 protein expression across prostate adenocarcinoma (PC-3, RWPE-1) and ovarian carcinoma (SKOV3, A2780, IOSE80) cell lines. Upper panel: replicate 1; lower panel: replicate 2. Target band: POFUT1 (~44 kDa); loading control: GAPDH (~36 kDa). Images are presented without cropping, brightness adjustment, or digital post-processing in compliance with raw data requirements.
