# Supplementary material for: Comprehensive Pan-Cancer Analysis Identifies POFUT1 as a Prognostic Biomarker and Potential Therapeutic Target Associated with Immune Evasions
Source: Cancers (Basel). 2026 Apr 23;18(9):1342. doi: 10.3390/cancers18091342 (PMC13163059; doi:10.3390/cancers18091342)
Supplement: Supplementary file 1 [file cancers-18-01342-s001.zip › cancers-4225350-supplementary.pdf]

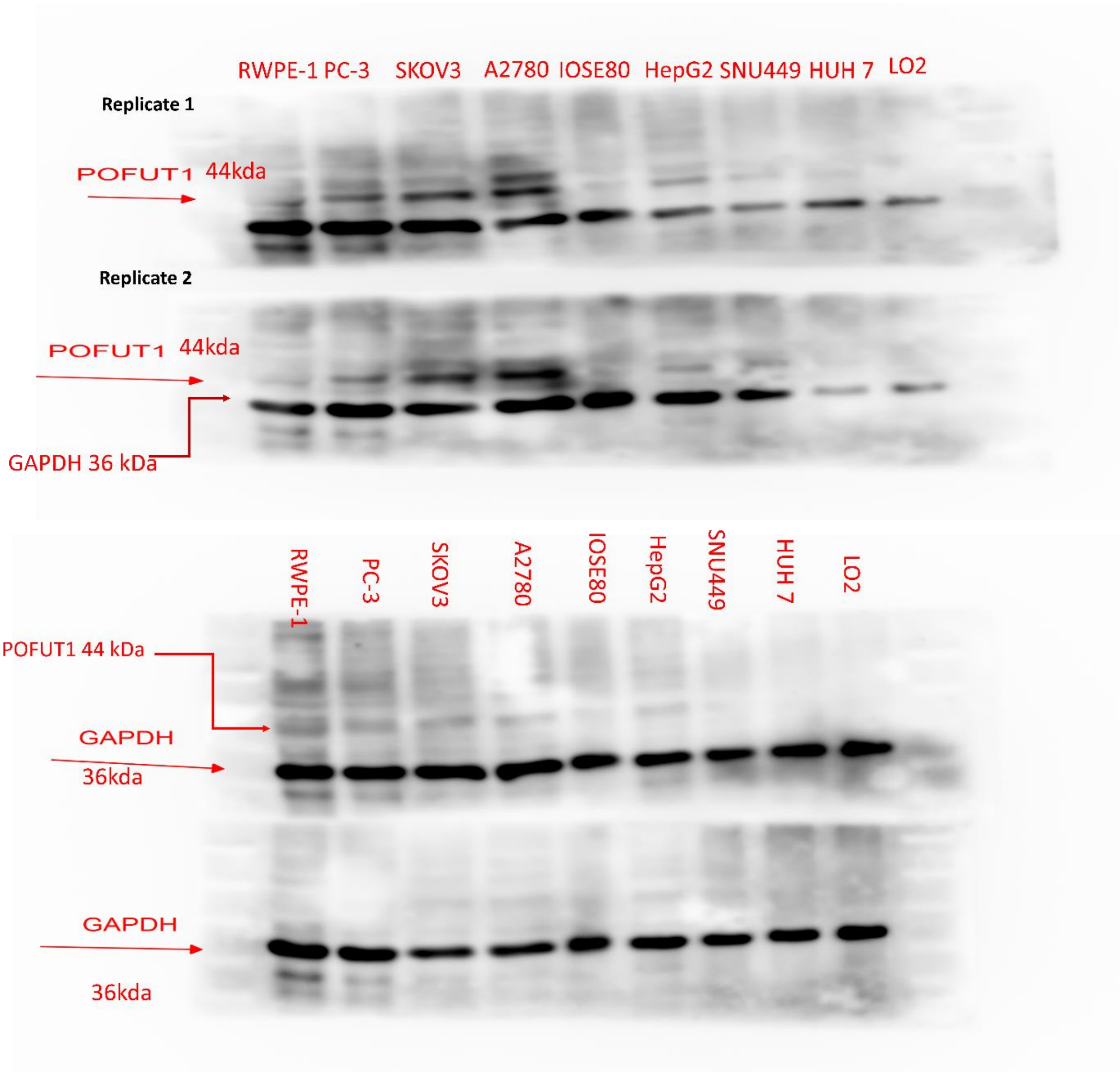

Supplementary Figure S1. Full, uncropped Western blot membrane images from two independent biological replicates confirming POFUT1 protein expression in prostate adenocarcinoma (PC-3, RWPE-1) and ovarian carcinoma (SKOV3, A2780, IOSE80) cell lines. Replicate 1 (upper panel) and Replicate 2 (lower panel) are shown. Bands corresponding to POFUT1 (~44 kDa) and GAPDH (~36 kDa) loading control are indicated. Images are presented without any cropping, brightness modification, or post-processing. Redundant membrane regions containing no target bands have been removed in the main text Figure 9H for visual clarity; full unprocessed images are shown here. HepG2/SNU449/HUH7/LO2 show variable POFUT1 expression consistent with the LIHC computational finding of context-dependent regulation
